# Multinormal *in vitro* distribution of *Plasmodium falciparum* susceptibility to piperaquine and pyronaridine

**DOI:** 10.1186/s12936-015-0586-6

**Published:** 2015-02-05

**Authors:** Aurélie Pascual, Marilyn Madamet, Sébastien Briolant, Tiphaine Gaillard, Rémy Amalvict, Nicolas Benoit, Dominique Travers, Bruno Pradines, the French National Reference Centre for Imported Malaria Study Group

**Affiliations:** Unité de Parasitologie, Département d’Infectiologie de Terrain, Institut de Recherche Biomédicale des Armées, Marseille, France; Aix Marseille Université, Unité de Recherche sur les Maladies Infectieuses et Tropicales Emergentes, UM 63, CNRS 7278, IRD 198, Inserm 1095, Marseille, France; Centre National de Référence du Paludisme, Marseille, France; Equipe Résidente de Recherche en Infectiologie Tropicale, Institut de Recherche Biomédicale des Armées, Hôpital d’Instruction des Armées Laveran, Marseille, France; Direction Inter-Armées du Service de Santé, Cayenne, Guyane France; Laboratoire de Parasitologie, Institut Pasteur de la Guyane, Cayenne, Guyane France; Fédération des Laboratoires, Hôpital d’Instruction des Armées Saint Anne, Toulon, France; Unité de Parasitologie et d’Entomologie, Département des Maladies Infectieuses, Institut de Recherche Biomédicale des Armées, Brétigny sur Orge, France

**Keywords:** Malaria, *Plasmodium falciparum*, Anti-malarial, *In vitro*, Resistance, Piperaquine, Pyronaridine

## Abstract

**Background:**

In 2002, the World Health Organization recommended that artemisinin-based combination therapy (ACT) be used to treat uncomplicated malaria. Dihydroartemisinin-piperaquine and artesunate-pyronaridine are two of these new combinations. The aim of the present work was to assess the distribution of the *in vitro* values of pyronaridine (PND) and piperaquine (PPQ) and to define a cut-off for reduced susceptibility for the two anti-malarial drugs.

**Methods:**

The distribution and range of the 50% inhibitory concentration values (IC_50_) of PND and PPQ were determined for 313 isolates obtained between 2008 and 2012 from patients hospitalized in France for imported malaria. The statistical Bayesian analysis was designed to answer the specific question of whether *Plasmodium falciparum* has different phenotypes of susceptibility to PND and PPQ.

**Results:**

The PND IC_50_ values ranged from 0.6 to 84.6 nM, with a geometric mean of 21.1 ± 16.0 nM (standard deviation). These values were classified into three components. The PPQ IC_50_ values ranged from 9.8 to 217.3 nM, and the geometric mean was 58.0 ± 34.5 nM. All 313 PPQ values were classified into four components. Isolates with IC_50_ values greater than 60 nM or four-fold greater than 3D7 IC_50_ are considered isolates that have reduced susceptibility to PND and those with IC_50_ values greater than 135 nM or 2.3-fold greater than 3D7 IC_50_ are considered isolates that have reduced susceptibility to PPQ.

**Conclusion:**

The existence of at least three phenotypes for PND and four phenotypes for PPQ was demonstrated. Based on the cut-off values, 18 isolates (5.8%) and 13 isolates (4.2%) demonstrated reduced susceptibility to PND and PPQ, respectively.

## Background

Over the past 20 years, many strains of *Plasmodium falciparum* have become resistant to chloroquine and other anti-malarial drugs. In 2002, the World Health Organization (WHO) recommended that artemisinin-based combination therapy (ACT) be used to treat all cases of uncomplicated malaria. The following combinations have been evaluated: artesunate-sulphadoxine-pyrimethamine, artesunate-amodiaquine, artemether-lumefantrine, artesunate-mefloquine, artesunate-chlorproguanil-dapsone and, more recently, artesunate-pyronaridine and dihydroartemisinin-piperaquine. Most of these combinations are available as fixed-dose co-formulations that are convenient, facilitate improved adherence and help prevent misuse.

Dihydroartemisinin-piperaquine (DP) (Artekin®, Duo-Cotecxin®, Eurartesim®) is a new ACT that is administered as single daily dose for three days. It has been demonstrated to be well tolerated and highly effective for the treatment of uncomplicated malaria in Asia [1,2] and for the treatment of uncomplicated *P. falciparum* malaria in Africa [3-6]. DP may also have a better post-treatment prophylactic effect than does artemether-lumefantrine [7-9] and artesunate-amodiaquine [10]. Since 2012, DP has been available for the treatment of uncomplicated falciparum malaria in France. DP has also been demonstrated to be effective for the treatment of *Plasmodium vivax* malaria [11]. However, the emergence of *P. falciparum* resistance to DP, manifested as delayed parasite clearance following the treatment, has developed in Cambodia and Vietnam [12-14].

The piperaquine (PPQ) susceptibility of *P. falciparum* isolates has been assessed in studies with isolates from Africa (geometric mean IC_50_ = 81.3 nM and 66.8 nM) [15,16], Cameroon (39 nM) [17], Kenya (from 41.9 to 50 nM) [18-20], Niger (24.2 nM) [21], Ghana (28.3 nM) [22], Uganda (6.1 nM) [23], the China-Myanmar border (28.4 nM) [24], the Thai-Burmese border (49 nM) [25], Cambodia (22 nM) [26], Indonesia (21.8 nM) [27], and Papua New Guinea [28].

The pyronaridine-artesunate combination (Pyramax®) is one of the latest artemisinin-based combinations and is currently under development by the not-for-profit organization Medicines for Malaria Venture (Geneva, Switzerland) and the pharmaceutical company Shin Poong Pharmaceuticals (Seoul, Republic of Korea) for the treatment of uncomplicated *P. falciparum* malaria and for the blood stages of *P. vivax* malaria. Pyramax® has recently completed phase III trials in humans. A five-day regimen of pyronaridine (PND) alone (total dose = 1,800 mg) produced a better cure rate than did artesunate, artemether or mefloquine used alone in the same conditions in Thailand [29]. The efficacy of PND-artesunate was not inferior to that of artemether-lumefantrine in the treatment of uncomplicated falciparum malaria in Africa and Southeast Asia [30,31]. PND-artesunate had a better efficacy than did mefloquine-artesunate in Cambodia [32].

The PND *in vitro* susceptibility was previously assessed in *P. falciparum* strains (1.9 to 47.8 nM and 15 to 49 nM, respectively) [33,34] and in isolates from Africa (geometric mean = 19.9 nM) [16], Cameroon (3.58 nM) [35], Gabon (3.0 nM and 1.87 nM) [36,37], Kenya (13.5 nM) [19], Niger (9.8 nM) [21], Senegal (3.8 nM and 4.52 nM) [38,39], Indonesia (1.92 nM) [40], and in isolates from patients in Thailand that were cured with PND (15.7 nM) or that recrudesced after PND treatment (23.0 nM) [29]. In addition, PND is effective *in vitro* against *P. vivax* isolates (2.58 nM) [40].

The early detection of resistance to PPQ and PND requires the establishment of the baseline parasite chemosusceptibility of current isolates from regions of endemicity. The aim of the present work was to determine the distribution and range of the 50% inhibitory concentrations (IC_50_) of PPQ and PND for 313 imported malaria isolates from Africa and to determine the cut-off values for *in vitro* reduced susceptibility to these two drugs.

## Methods

### Patients and sample collection

In total, 313 *P. falciparum* isolates were collected between April 2008 and August 2012 from patients hospitalized in France with imported malaria from a malaria-endemic country (Angola, Benin, Burkina Faso, Cameroon, Central African Republic, Chad, Comoros, Congo, Ivory Coast, Gabon, Gambia, Ghana, Guinea, Madagascar, Mali, Mauritania, Mozambique, Niger, Senegal, Togo, Zambia). Informed consent was not required for this study because the sampling procedures and testing are part of the French national recommendations for the care and surveillance of malaria. Venous blood samples were collected in Vacutainer® ACD tubes (Becton Dickinson, Rutherford, NJ, USA) before treatment and were transported at 4°C from French hospitals located in Aix en Provence, Bordeaux, Chambery, Frejus, Grenoble, Lyon, Marseille, Metz, Montpellier, Nice, Nimes, Pau, Toulon, Toulouse, and Valence to the Institute of Biomedical Research of the French Army (IRBA) in Marseille within 72 hours of collection. A Case Report Form was provided at the same time, either as a paper copy or electronically.

Thin blood smears were stained using a RAL® kit (Réactifs RAL, Paris, France) and were examined to determine the *P. falciparum* density and confirm mono-infection. Parasitized erythrocytes were washed three times with RPMI 1640 medium (Invitrogen, Paisley, UK), buffered with 25 mM HEPES and 25 mM NaHCO_3_. If the parasitaemia exceeded 0.5%, the infected erythrocytes were diluted to 0.5% with uninfected erythrocytes (human blood type A+) and re-suspended in RPMI 1640 medium supplemented with 10% human serum (Abcys S.A. Paris, France) for a final haematocrit of 1.5%. The susceptibility of the 313 isolates was assessed without culture adaptation.

### Drugs

PPQ and PND were obtained from Shin Poong Pharm Co. (Seoul, Korea). PPQ was first dissolved in methanol and then diluted in water to obtain final concentrations ranging from 0.8 to 1,000 nM. PND was dissolved and diluted in water to obtain concentrations ranging from 0.15 to 100 nM.

Batches of plates were tested and validated using the chloroquine-susceptible strain 3D7 (isolated in West Africa; obtained from MR4, VA, USA) in three to six independent experiments using the conditions described below. The two strains were synchronized twice with sorbitol before use [41], and clonality was verified every 15 days through PCR genotyping of the polymorphic genetic markers *msp1* and *msp2* and microsatellite loci [42,43]; additionally, clonality was verified each year by an independent laboratory from the Worldwide Anti-malarial Resistance Network (WWARN).

### *Ex vivo* assay

For *ex vivo* isotopic microtests, 200 μl/well of the suspension of parasitized red blood cells (final parasitaemia, 0.5%; final haematocrit, 1.5%) were distributed in 96-well plates pre-dosed with anti-malarial drugs. Parasite growth was assessed by adding 1 μCi of tritiated hypoxanthine with a specific activity of 14.1 Ci/mmol (Perkin-Elmer, Courtaboeuf, France) to each well at time zero. The plates were then incubated for 42 hours in controlled atmospheric conditions that consisted of 10% O_2_, 5% CO_2_ and 85% N_2_ at 37°C with a humidity of 95%. Immediately after incubation, the plates were frozen and then thawed to lyse the erythrocytes. The contents of each well were collected on standard filter microplates (Unifilter GF/B; Perkin-Elmer) and washed using a cell harvester (Filter-Mate Cell Harvester; Perkin-Elmer). The filter microplates were dried, and 25 μl of scintillation cocktail (Microscint O; Perkin-Elmer) was placed in each well. Radioactivity incorporated in nucleotides by the parasites was measured with a scintillation counter (Top Count; Perkin-Elmer).

The drug concentration able to inhibit 50% of the parasite growth (IC_50_) was assessed by the drug concentration corresponding to 50% of the incorporation of tritiated hypoxanthine by the parasite in the drug-free control wells. The IC_50_ value was determined using a non-linear regression analysis of log-based dose-response curves (Riasmart, Packard, Meriden, USA).

### Statistical analysis

The statistical analysis was designed to answer the specific question of whether *P. falciparum* has different PPQ and PND susceptibility phenotypes. A heterogeneous population of IC_50_ values was observed; therefore, the data were assumed to represent a univariate Gaussian mixture with k components. Each observation was assumed to originate from one of the k components, and the label of the group from which each observation arose was unknown. The unknowns of the model were the number of components, the means, variances and weights of the different components, and the vector of allocations of the observations. The analysis was performed in two steps. First, reversible jump Monte Carlo Markov Chains (RJMCMC) [44] samplers were used to choose a suitable number of components k, and the present algorithm followed the recommendations of Cappé *et al.* [45]. After a relevant number of components was chosen, standard Gibbs samplers were run to obtain estimates of the model parameters and to classify the observations [46]. Because of the ‘label-switching’ problem that is due to the symmetry in the likelihood of the model parameters, the mixture components should be labelled before making an inference regarding the parameters [47]. The classical ordering constraint, which was biologically relevant here, was used. The algorithms were run for 100,000 burn-in iterations and 20,000 post-burn-in iterations. These numbers were assumed to be sufficient to obtain reliable results. Moreover, each algorithm was run three times to verify that the results obtained in two different runs were similar and that there was no convergence problem [44].

## Results

**Figure 1:**
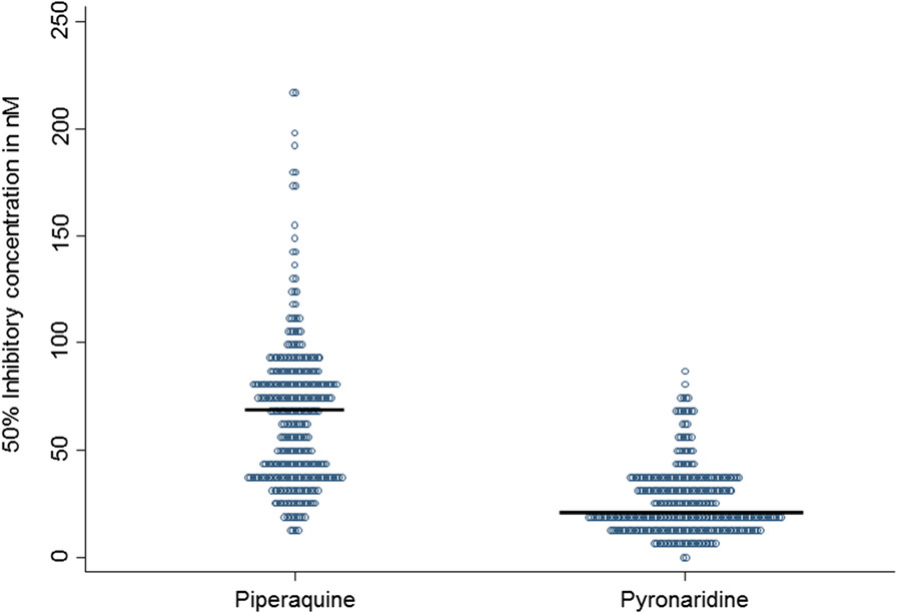
**Piperaquine and pyronaridine median 50% inhibitory concentrations (IC50 values in nM) of 313** ***Plasmodium falciparum*** **isolates.**

The PND IC_50_ values ranged from 0.6 to 84.6 nM (Figure 1). The geometric mean was 21.1 ± 16.0 nM (standard deviation). The average parameter estimates for the IC_50_ values by year are given in Table 1. There was no significant difference in the PND responses between the five years (p = 0.9416, Kruskal-Wallis rank sum test). In addition, there was no significant difference in the responses to PND against the strain 3D7, which was used as a control for the plate batches (p = 0.8904).

**Table 1:** **Statistical analysis of the 309 pyronaridine (PND) IC**_**50**_ **values by year**

**Year**	**IC**_**50**_ **number**	**Mean (nM)**	**SD**	**IC**_**50**_ **min**	**IC**_**50**_ **max**
2008	60	21.4	14.5	3.8	67.4
2009	92	21.9	15.2	3.2	76.1
2010	88	19.4	15.7	0.6	80.0
2011	47	23.0	18.3	6.2	75.3
2012	22	21.1	19.3	9.1	84.6
Total	309	21.1	16.0	0.6	84.6

SD: standard deviation.

**Figure 2:**
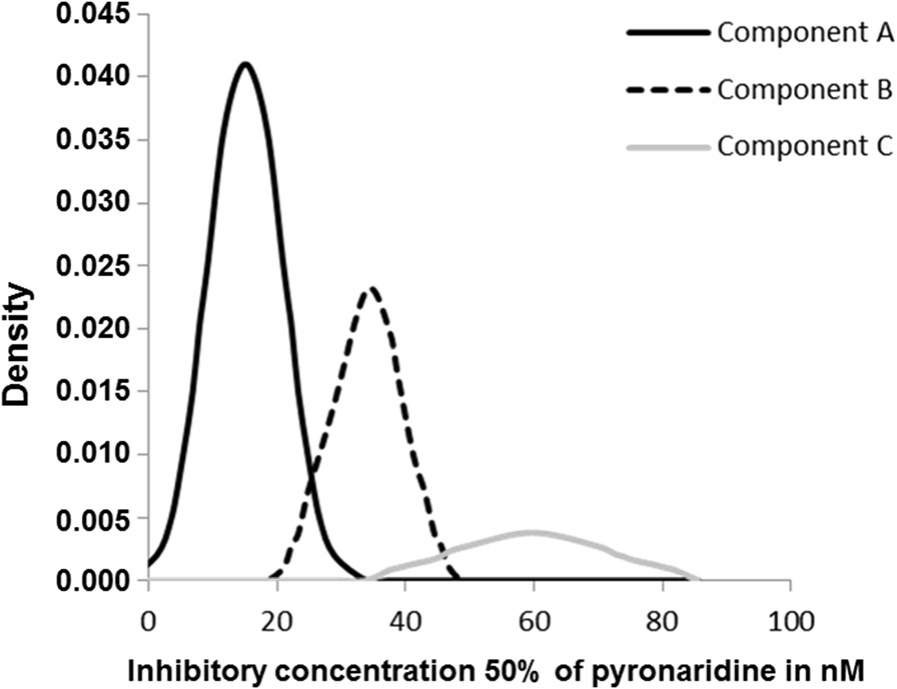
**Distribution of the pyronaridine IC**_**50**_ **values of the 309** ***Plasmodium falciparum*** **isolates in the three-component mixture model (Bayesian mixture modelling approach).** The dotted lines represent the three fitted mixtures.

The triple normal distribution model for PND is represented in Figure 2. The parameter estimates for the three-component mixture model, including the number of isolates in each normal distribution, the mean of the IC_50_ values and the standard deviation (SD) for each distribution, are summarized in Table 2.

**Table 2:** **Parameter estimates for the three-component mixture model for the pyronaridine (PND) distribution of the 309** ***Plasmodium falciparum*** **isolates**

**Component**	**Isolates number**	**Proportion (%)**	**IC**_**50**_ **mean (nM)**	**Standard deviation**
A	182	58.9	15.1	5.6
B	96	31.1	33.9	5.3
C	31	10.0	59.7	12.2

The cut-off value for *in vitro* reduced susceptibility to PND was estimated using the arithmetic mean plus two SDs of the IC_50_s of the 309 isolates and was set at 57.7 nM. Isolates with an IC_50_ greater than 60 nM were considered to display reduced susceptibility to PND *in vitro*. Eighteen isolates (5.8%) displayed reduced susceptibility to PND *in vitro*.

The PPQ IC_50_ values ranged from 9.8 to 217.3 nM (Figure 1). The geometric mean was 58.0 ± 34.5 nM (standard deviation). The average parameter estimates for the IC_50_ values by year are given in Table 3. There was a significant difference in the PPQ responses between the five years (p <0.0001, Kruskal-Wallis rank sum test). However, there was no significant difference in the responses to PPQ against the strain 3D7, which was used as a control for the plate batches (p = 0.6909).

**Table 3:** **Statistical analysis of the 313 piperaquine (PPQ) IC**_**50**_ **values by year**

**Year**	**IC**_**50**_ **number**	**Mean (nM)**	**SD**	**IC**_**50**_ **min**	**IC**_**50**_ **max**
2008	60	77.8	40.5	20.6	217.3
2009	95	70.6	30.6	14.0	189.0
2010	89	44.0	23.4	11.8	92.3
2011	47	44.6	20.9	11.8	123.0
2012	22	59.7	46.2	9.8	196.0
Total	313	58.0	34.5	9.8	217.3

SD: standard deviation.

**Figure 3:**
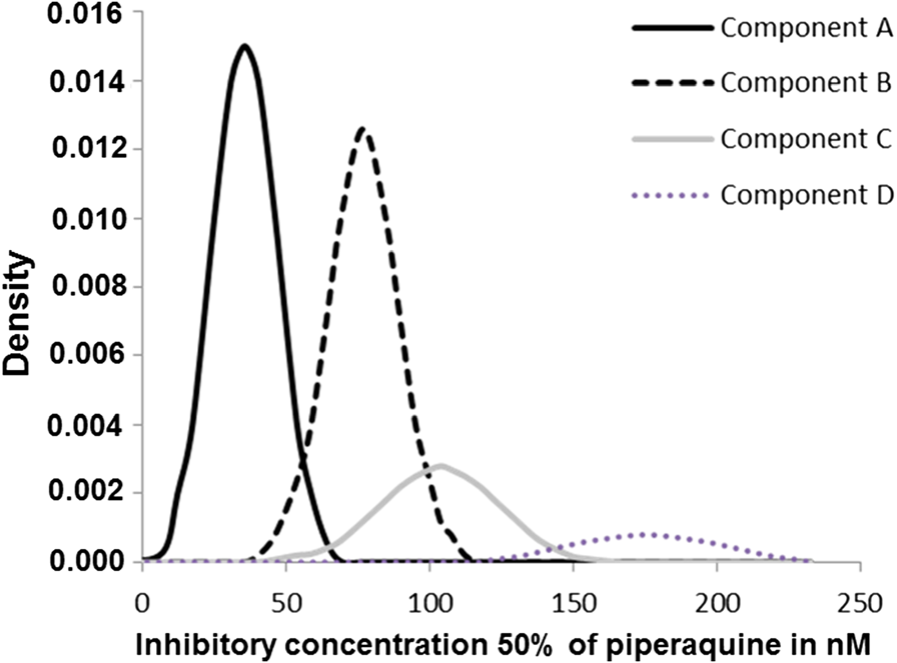
**Distribution of the piperaquine IC**_**50**_ **values of the 313** ***Plasmodium falciparum*** **isolates in the four-component mixture model (Bayesian mixture modelling approach).** The dotted lines represent the three fitted mixtures.

The quadruple normal distribution model for PPQ is presented in Figure 3. The parameter estimates for the quadruple-component mixture model, including the number of isolates in each normal distribution, the mean of the IC_50_ values and the standard deviation for each distribution, are summarized in Table 4.

**Table 4:** **Parameter estimates for the four-component mixture model for the piperaquine PPQ) distribution of the 313** ***Plasmodium falciparum*** **isolates**

**Component**	**Isolates number**	**Proportion (%)**	**IC**_**50**_ **mean (nM)**	**Standard deviation**
A	136	43.5	35.4	11.4
B	146	46.6	76.7	12.6
C	22	7.0	103.5	20.4
D	9	2.9	175.0	26.2

The cut-off for *in vitro* reduced susceptibility to PPQ was estimated using the arithmetic mean plus 2 SDs of the IC_50_s of the 313 isolates and was found to be 135.4 nM. Isolates with an IC_50_ greater than 135 nM were considered to be isolates with reduced susceptibility to PPQ *in vitro*. Thirteen isolates (4.2%) displayed reduced susceptibility to PPQ *in vitro*.

## Discussion

The early detection of resistance to PPQ and PND requires that the baseline parasite chemosusceptibility of current isolates from regions of endemicity be established. Maximizing the efficacy and longevity of drugs as a tool to control malaria will critically depend on pursuing intensive research into identifying *in vitro* markers and implementing *in vitro* and *in vivo* surveillance programmes, such as those championed by WWARN [48,49]. In this context, there is a need to identify *in vitro* and molecular markers that predict PPQ and PND resistance and can provide an active surveillance method to monitor temporal trends in parasite susceptibility [50,51].

A Bayesian mixture modeling approach was choose. A Bayesian approach has already been proposed for anti-malarial *in vitro* susceptibilities; it has been used to evaluate the distribution and the cut-off for reduced susceptibility to doxycycline [52,53]. All 309 PND values were classified into three components: component A (IC_50_ mean 15.1 nM), component B (IC_50_ mean 33.9 nM) and component C (IC_50_ mean 59.7 nM). The proportion of isolates in each group was 58.9% for component A, 31.1% for component B and 10.0% for component C. In the previous study on doxycycline, the distribution of the doxycycline IC_50_ in a triple normal distribution was independent of the African origin of the isolates (imported isolates from Africa or field isolates from Senegal, Gabon or Congo) [52]. Only the proportion of isolates predicted to belong to each component was dependent on the origin of the isolates.

In the present study, the cut-off for *in vitro* reduced susceptibility to PND was estimated as the geometric mean plus 2 SDs of the IC_50_s of the 309 isolates (57.7 nM). Isolates with an IC_50_ greater than 60 nM were considered to be isolates with reduced *in vitro* susceptibility to PND. Eighteen isolates (5.8%) demonstrated reduced susceptibility to PND *in vitro*. These data are consistent with clinical observations of limited failures with PND. One study of isolates in Niger reported that 3% of the isolates displayed *in vitro* resistance to PND, with an estimated cut-off of 20 nM [21].

A cut-off for *in vitro* resistance is defined for a specific methodology. For example, the *in vitro* effects and the IC_50_ values for doxycycline are dependent on the time incubation conditions [14-16], on gas conditions, i.e., O_2_ and CO_2_ [54,55] and on methodology, i.e., isotopic test *versus* immuno-enzymatic or SYBR green test [56,57]. The incubation time is the condition that interferes significantly with the IC_50_ values for doxycycline or other antibiotics [58,59]. The IC_50_ values decrease by a factor between 10 to 100 in prolonged exposure to antibiotics. The gas conditions interfere with the IC_50_ values for quinolines, such as chloroquine, monodesethyaodiaquine, quinine, mefloquine, or lumefantrine [55,60]. The IC_50_ values for quinoline drugs are significantly lower at O_2_ > 15% than those at 10% O_2_. Dissolution methods to prepare the stock solutions of anti-malarial drugs can also interfere with IC_50_ values. In the present study, the dissolution of PND in water was the same condition as this used in previous work [16,36,37,61]. Using an arbitrarily fixed threshold of resistance could lead to wrong decision making at the country level. To reduce the effects of the conditions of the methodology of the *in vitro* test, an IC_50_ ratio (IC_50_ of clinical isolate/mean IC_50_ of 3D7 on the same batch of plates) can be evaluated for each isolate [56]. Another mean is to use a cut-off ratio (resistance cut-off defined for a specific methodology/mean IC_50_ of 3D7 tested by the same methodology). Isolates with cut-off ratio greater than four (60/16.7), i.e., isolates with IC_50_ four-fold greater than 3D7 IC_50_, are considered to be isolates with reduced *in vitro* susceptibility to PND. Compared to 3D7 IC_50_ values, the data obtained with different methodologies can be compared.

All 313 PPQ values were classified into four components: component A (IC_50_ mean 35.4 nM), component B (IC_50_ mean 76.7 nM), component C (IC_50_ mean 103.5 nM), and component D (IC_50_ mean 175.0 nM). The proportion of isolates in each group was 43.5% for component A, 46.6% for component B, 7.0% for component C, and 2.9% for component D.

In the present study, PPQ was dissolved in methanol and then diluted in water. In previous works, PPQ was dissolved in methanol and then diluted in water [13,14,24,25], in methanol and hydrochloric acid and then diluted in water [20,62], in dimethyl sulphoxide (DMSO) [27,63] or in 0.5% acid lactic in water [15,28,63-65]. These several dissolution methods to prepare the PPQ stock solutions can interfere with IC_50_ values. The cut-off for *in vitro* reduced susceptibility to PPQ was estimated to be the geometric mean plus 2 SDs of the IC_50_s of the 313 isolates (135.4 nM). Isolates with an IC_50_ greater than 135 nM or 2.3-fold greater than PPQ IC_50_ for 3D7 were considered to be isolates with reduced *in vitro* susceptibility to PPQ. Thirteen isolates (4.2%) met the criteria for reduced susceptibility to PPQ *in vitro*. These data are consistent with previous data from Niger but not from the China-Myanmar border. In Niger, 6% of the isolates were found to be resistant to PPQ *in vitro*, with an estimated cut-off of 150 nM [21]. However, 83% of the isolates from the China-Myanmar border were resistant to PPQ *in vitro* (the cut-off of approximately 15 nM was estimated by a three-fold decrease in susceptibility to PPQ in comparison to the strain 3D7) [24].

There was a significant difference in the PPQ responses among the five years of the study (p <0.0001, Kruskal-Wallis rank sum test). However, there was no significant difference in the responses to PPQ against 3D7 (geometric mean each year from 2008 to 2012: 56.7 nM, 58.4 nM, 61.0, 57.2 nM, and 62.5 nM; p = 0.6909). This absence of significant difference in the responses of the control strains makes bias due to the methodology unlikely.

The existence of at least three phenotypes for PND and four phenotypes for PPQ was demonstrated. These phenotypes may be associated with different genotypes. Genotyping analysis would be necessary to identify the molecular basis of the susceptibility differences and to correlate the genetic profiles with the phenotypes. Previous work demonstrated that the doxycycline phenotypes predicted by the Bayesian method were associated with specific genotypes [53,63]. The priority now is to investigate polymorphisms both in the genes that are known to be involved in anti-malarial drug resistance and in new genes for each phenotype.

## Conclusion

The PND and PPQ *in vitro* susceptibility values ranged into three and four components, respectively. Eighteen isolates (5.8%) and 13 isolates (4.2%) demonstrated reduced *in vitro* susceptibility to PND and PPQ, respectively. DP is associated with a longer prophylactic time after treatment compared to the time after artemether-lumefantrine treatment [5,6,64]. However, the gametocyte carriage and malaria transmission to mosquitoes was lower after artemether-lumefantrine treatment [66]. Despite the recent report of frequent DP failures in Cambodia and Vietnam [12-14], DP remains an effective treatment for falciparum malaria in Africa and for *P. vivax* [67-69]. PND-artesunate is effective for the treatment of uncomplicated falciparum malaria in Africa and Southeast Asia [30,31]. PND-artesunate successfully treats artemisinin-resistant *Plasmodium berghei* parasites, while artemether-lumefantrine, artesunate-amodiaquine, artesunate-mefloquine and dihydroartemisinin-piperaquine are not effective [70]. Although extended parasite clearance times were indicative of artemisinin resistance, ACT remains important for the treatment of malaria.
